# Active Antialopecia Chemical Identification of *Merremia peltata* Leaves and Computational Study toward Androgen Receptor Using Molecular Docking and Molecular Dynamic Simulation

**DOI:** 10.1155/2022/1123047

**Published:** 2022-08-08

**Authors:** Syawal Abdurrahman, Ruslin Ruslin, Aliya N. Hasanah, Resmi Mustarichie, Mus Ifaya

**Affiliations:** ^1^Department of Pharmaceutical Analysis and Medicinal Chemistry, Faculty of Pharmacy, Universitas Padjadjaran, Andir 45363, Indonesia; ^2^Department of Medical Laboratory Technology, Universitas Mandala Waluya, Kendari 93231, Indonesia; ^3^Department of Medicinal Chemistry, Faculty of Pharmacy, Universitas Halu Oleo, Kendari 93231, Indonesia; ^4^Department of Pharmacy, Universitas Mandala Waluya, Kendari 93231, Indonesia

## Abstract

Alopecia is a health condition in which the hair loses its function in some or all of the body. Alopecia occurs due to various genetic, environmental, and nutritional factors. One of the methods developed to treat alopecia is through inhibition of the enzyme 5-*α*-reductase, which converts testosterone into its more potent metabolite, dihydrotestosterone (DHT). In ethnomedicine, the leaves of *Merremia peltata* are used by the people of Sulawesi as a remedy for baldness. Therefore, in this study, an in vivo study was conducted on rabbits to investigate the antialopecia activity of the ethanolic extract of *M. peltata* leaves. The purified *M. peltata* leaf extract was fractionated using vacuum liquid chromatography with several solvents to produce fractions F1–F5. Each fraction was then retested in vivo in rabbits, and its content was then analyzed by LC-MS. An in silico study was then carried out using minoxidil as a comparison ligand; 17 compounds derived from *M. peltata* leaves were identified as antialopecia compounds through prediction of molecular interactions and molecular dynamics simulation and prediction of absorption, distribution, metabolism, excretion, and toxicology (ADME-Tox). The assay results showed that fractions F2 and F3 had a better effect on hair growth compared to the positive control, and the test compound obtained from the LC-MS analysis, bufotalinin, had a strong binding energy to the receptor in the molecular docking interaction study: −5.99 kcal/mol compared to −4.8 kcal/mol for minoxidil. Molecular dynamics simulation analysis with complex stability parameters based on solvent-accessible surface area (SASA), principal component analysis (PCA), root mean square deviation (RMSD), and root mean square fluctuation (RMSF) showed that bufotalinin has good affinity for androgen receptors. ADME-Tox prediction for bufotalinin showed good results for the parameters of skin permeability, absorption, and distribution. Therefore, bufotalinin, a steroid compound, is a potential androgen receptor antagonist and could be useful in the treatment of alopecia.

## 1. Introduction

Alopecia is a medical term that refers to hair loss. Alopecia causes hair to lose its function, and this abnormality will occur on the scalp and can extend to all areas of the body [1, 2]. In 2014, alopecia was experienced by 35 million men and 21 million women worldwide; 66% of men and 40% of women who experienced alopecia were over the age of 35 years. Alopecia can be caused by genetic, environmental, and nutritional factors. Nutrients in the form of protein and vitamins contained in hair, such as vitamins A, D, E, and B12, play an important role in hair maintenance [3]. One of the efforts to prevent baldness is through inhibition of the enzyme 5-*α*-reductase using a synthetic drug in the form of minoxidil; but continuous topical treatment with minoxidil can cause side effects such as hypertrichosis. In addition, it is expensive and does not cure hair loss as a whole [2]. In addition to using synthetic drugs, various other methods can be used to treat alopecia, one of which is the use of compounds contained in the plant *Merremia peltata* originating from Indonesia. Ethnobotanically, this plant has been used by the people of Konawe, Southeast Sulawesi, to treat dandruff and hair growth [4]. The secondary metabolites contained in the leaves of *M. peltata* include terpenoids, steroids, saponins, alkaloids, and flavonoids [5, 6]. According to [7], compounds that play an active role in the process of hair fertilization are alkaloids, pyrones, and flavonoids. Derivatives of flavonoid compounds from the flavanone group are known to affect hair growth through therapeutic methods. [8] determined *in silico* that flavonoid compounds have antialopecia activity. According to [3], vitamin B3 and biotin, which are derivatives of alkaloid compounds, can act as alternatives in preventing hair loss.

They stated that glycyrrhizin compounds derived from compounds belonging to the phytosterol group inhibit 5-*α*-reductase in the formation of dihydrotestosterone compounds that cause alopecia and have advantages in the treatment of Androgenetic alopecia (AGA) [9]. Epigallocatechin gallate, a compound contained in green tea, according to [10], showed significant results with increased hair growth. [11] showed, in clinical trials, that procyanidin B2 compounds contained in apple extract can increase hair growth and scalp quality, as well as increase the density, weight, and keratin content of hair. *In silico* studies carried out by [12] have shown that the terpenoid and steroid compounds contained in *M. peltata* leaves have antialopecia activity based on the docking results for androgen receptors. The leaves of *M. peltata* contain steroids, one form of which is the hormone. Testosterone can trigger hair growth in men; the presence of 5*α*-reductase will inhibit the work of the testosterone, which plays a role in the anagen phase of the hair growth cycle. 5*α*-Reductase works by reducing the vinyl group contained in the first aliphatic ring on the structure of testosterone that produces a single bond at the C*α* position to form the compound known as dihydrotestosterone (DHT). Based on previous research, in this study, pharmacological tests were carried out on the extracts and fractions of *M. peltata* leaf obtained by vacuum liquid chromatography (VLC), which aimed to investigate its antialopecia activity and identify the secondary metabolites in the *M. peltata* leaf active fraction using liquid chromatography–mass spectrometry (LC-MS/MS). Furthermore, interaction stability, molecular docking, and molecular dynamics (MD) were analyzed to determine the molecular interaction of the compounds identified in the active fraction using LC-MS/MS with the target protein.

## 2. Methods

### 2.1. Sample Preparation and Determination

*M. peltata* leaves were obtained from the Rawa Aopa National Park, Southeast Sulawesi, and the determination of the *M. peltata* leaves (844/I1.CO2.2/PL/2020) was carried out at the Herbarium of the School of Life Sciences Technology, Bandung Institute of Technology, West Java.

### 2.2. Extraction

Dried powder of *M. peltata* leaves was obtained from the preparation of 1 kg of samples, which was subjected to extraction by maceration using 96% ethanol solvent for 3 × 24 h. The ethanol extract of *M. peltata* leaves in viscous form was 244.86 g, a yield of 24.486%.

### 2.3. Purified Extract

The chlorophyll contained in the ethanol extract was removed by liquid–liquid extraction using a methanol water ratio of 1 : 1; the mixture was stirred and allowed to stand for 24 h until a precipitate was formed. The precipitate in the form of chlorophyll was then separated from the filtrate. The chlorophyll-free filtrate (3 L) was partitioned with ethyl acetate (1 : 1). MgSO4 powder was then added to the ethyl acetate phase as a drying agent and filtered. The ethyl acetate phase was concentrated, and 40 g of ethyl acetate extract was obtained. Separation analysis was performed using thin layer chromatography (TLC) with n-hexane : ethyl acetate (1 : 1) as eluent to determine the separation pattern of each purified fraction [13].

### 2.4. Hair Growth Activity Assay

A hair growth activity test was carried out using the ethyl acetate extract of *M. peltata* leaves at concentrations of 10%, 20%, and 30%. The method of [14] used in this test is one of the methods for determining hair growth activity by shaving; the back of the rabbit's hair is shaved clean and then divided into six 2 × 2 cm squares. The test used six healthy male rabbits (number calculated by the rule of [15] aged 4–5 months, with no anatomical defects. Prior to use, rabbits were acclimatized for 7 days. Then, the fur was removed from the back of the rabbit, and it was rested for 24 h. After that, the test material was applied to the test area once a day for 21 days. The first day of application is considered as day 0. The test was carried out in two stages. The first test stage was carried out to find the effective dose at the best extract concentration for rabbit hair growth. The test area was divided into six parts: positive control–smeared with 2% minoxidil; negative control–treated with Na-CMC; normal control without treatment; test groups–treated with 10%, 20%, and 30% ethanol extract of *M. peltata* leaves, respectively. The second step was to find the most effective dose of the extract fractions obtained by VLC. In this treatment, the back of the rabbit was divided into eight parts: positive control: smeared with 2% minoxidil solution; negative control: treated with Na-CMC solution; normal control without treatment; test groups: treated with five extract fractions obtained by VLC, coded F1–F5. After obtaining data from the results of the study, data processing was carried out using statistical analysis of variance (ANOVA) [16].

### 2.5. Fractionation by VLC

The fractionation process was carried out by packing silica in a chromatographic column. Ethyl acetate extract of *M. peltata* leaves (20 g) was put into a chromatographic column with a silica gel stationary phase and separated using n-hexane eluent: EtOAc in successive ratios of 8 : 2 (twice); 7 : 3 (twice); 5 : 5 (twice); 3 : 7 (twice); 100% EtOAc (twice); and 100% MeOH (twice). Separation by VLC resulted in five fractions, namely, F1–F5; collection of the fractions was based on the TLC pattern using n-hexane eluent: EtOAc (6 : 4). Analysis of the separation results was carried out using 254 and 366 nm UV lamps and reagents for cerium sulfate staining to determine the distribution of compounds contained in the purified ethyl acetate extract of *M. peltata* leaves.

### 2.6. Identification of Compounds Using LC-MS/MS

The chemical constituents of the fractions (F1–F5) obtained by VLC of the purified ethyl acetate extract of *M. peltata* leaves were identified by LC-MS/MS using an HPLC connected to a Q-TOF spectrometer equipped with a positive ion mode ESI source. A full scan was performed from m/z 100 to 1200 at a source temperature of 140°C. A Phenomenon HPLC column (5*μ*·C8; 150 × 2 mm i.d.) was used for analysis, with 0.3% formic acid methanol as the mobile phase with a flow rate of 0.1 mL/min using an isocratic system. The drying gas temperature (N2) in MS was 350°C, the gas flow rate was 6 mL/min, and the nebulization pressure (N2) was 25 psi. The sample (0.5 g) was diluted with methanol and filtered through a 0.22 m nylon filter prior to analysis. MS analysis was performed to determine the mass fragmentation (m/z).). The results of the MS spectrum were then compared with the similarity index (SI) in the LC-MS library by using spectrum database for organic compounds in SDBS application from AIST Japan [17]. The data obtained were processed by using UNIFI software (version 1.8, Waters Corporation, Milford, MA, USA) with a screening solution workflow, which helped in automated data processing for reporting the positive identifications. The result was compared with database that collected more than 1200 compounds based on chemical structure, molecular formula, and molecular mass from various web-based resources.

### 2.7. Preparation of Ligand Structure

The structure of the 17 test compounds obtained from the purified ethyl acetate extract of *M. peltata* leaves by VLC fractionation, as well as that of reference ligands minoxidil and finasteride, is shown in Table 1. Minoxidil, finasteride, and the test ligands were made into two-dimensional (2D) structures, which were then converted into a three-dimensional (3D) structure using ChemDraw 8.0.

### 2.8. Preparation of Protein Receptor

The crystal structure of the androgen receptor (4K7A) obtained from the PDB database (http/www/pdbbeta.rscb.org/pdb) was selected in the active form, which binds to natural ligands DHT and minoxidil with a resolution of 2.44; *Homo sapiens* was selected as the type of organism, and the mutation value was set to zero. Minoxidil is a natural ligand of the androgen receptor. DHT is a natural ligand that causes baldness, and minoxidil is a drug that is known to treat baldness. The 3D structure of the protein was downloaded into the PDB file format. Preparation of the androgen receptor (4K7A) and natural ligands was carried out using AutoDock Tools 1.5.6 to obtain the position of the grid box to determine the spatial shape and spatial coordinates as the docking material [18]. The position of the minoxidil grid box in the androgen receptor crystal structure was used as the position for docking analysis of all test ligands.

### 2.9. Validation of the Molecular Docking Method

Molecular docking validation is carried out using AutoDock Tools 1.5.6 by redocking a natural ligand against a target protein that has had its natural ligand removed. Here, validation was carried out by redocking the natural ligand minoxidil on the androgen receptor 4K7A; residues on the protein in the form of DHT, minoxidil, and water ligands contained in the protein receptor were removed by adjusting the position of the grid box o4n the natural ligand, minoxidil, which was then docked. The process was carried out to determine the RMSD value; natural ligand redocking is considered successful if the RMSD value obtained is < 3 Å [19]. An RMSD value of 2.31 Å was obtained by looking at the overlay of the natural ligand minoxidil, which was separated before docking and the redocked natural ligand minoxidil using Discovery Studio Visualizer.

### 2.10. Docking Simulation of Minoxidil, Finasteride, and Test Ligands (Phytocompounds in Purified *M. peltata* Leaf Extract)

Optimization of the 3D structure of minoxidil, finasteride, and test ligands was carried out using Chem3D Ultra 8.0 and the MM2 semiempirical computational method. The calculation is done by optimizing the geometry at the minimum energy of the 3D structure.

The docking method is done by tethering each ligand to the androgen receptor with pdbqt format using tether coordinates (Grid Center) *x* = 40, *y* = 40, *z* = 40 and the coordinates of Grid Box size *x* = −2.592, *y* = 0.864, *z* = −6.729. Each ligand is in a stable state, and interactions with biomacromolecules are in a rigid state.

The docking results obtained in the form of binding energy values and chemical interactions such as hydrogen bonds, hydrophobic interactions, and bond distances were visualized with Discovery Studio Visualizer [20].

### 2.11. MD Simulation

The MD simulation was carried out using bufotalinin, the ligand with the lowest binding energy to the androgen receptor, and GROMACS 2016.3 software with the AMBER99SB-ILDN force field [21]. Topology and ligand parameters were made using ACPYPE [22]. The electrostatic force over a distance was determined by the particle mesh Ewald method [23]. Neutralization of the system was carried out by adding Na+ and Cl− ions. Solvation was carried out using the TIP3P water cube model [24]. The simulation preparation stage includes the minimization step, heating to 310 K, temperature equilibration and pressure equilibration. Furthermore, 100 ns of MD production was performed with a 2 fs timestep. After the simulation, g_rms, g_rmsf, and g_rg functions were calculated. Post-MD simulation analysis was done by calculating the SASA and PCA to detect the direction and amplitude of the dominant motions, RMSD, and RMSF.

### 2.12. Prediction of ADME-Tox

The ADME-Tox SAR program can be accessed at http://biosig.unimelb.edu.au/pkcsm/prediction [25]. The structure of the resulting compound was converted into SMILE format using PubChem. The obtained structure was downloaded using the SMILE canonical link.

## 3. Results and Discussion

### 3.1. Hair Growth Activity of *M. peltata* Leaf Extract

**Figure 1:**
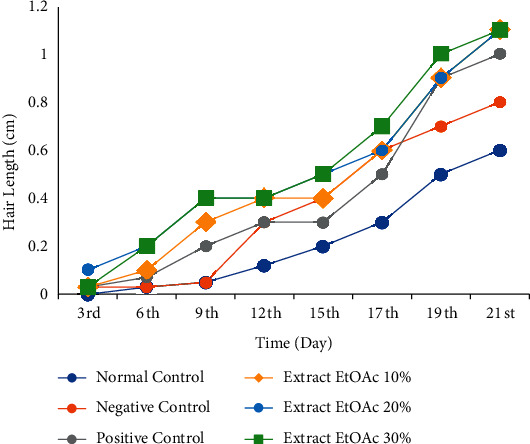
Test of *M. peltata* leaf ethyl acetate extract on rabbit hair growth.

An *in vivo* hair growth activity test of the ethyl acetate extract of *M. peltata* leaves at concentrations of 10%, 20%, and 30%, with a positive control of 2% minoxidil, a negative control of Na-CMC, and a normal control, was carried out for 21 days of observation on test animals. On average, the fasting hair growth level in the positive control (minoxidil) was 1.0 ± 0.2 mg/dL, in the normal control group, it was 0.6 ± 0.2 mg/dL, and in the negative control, it was 0.8 ± 0.2 mg/dL; the ethyl acetate extract of *M. peltata* leaves showed activity at 10%, 20%, and 30% at 1.1 ± 0.3, 1.1 ± 0.4, and 1.3 ± 0.2 mg/dL, respectively. The graph of the results of measuring the average hair growth in rabbits is seen in Figure 1.

The hair growth stimulation test of *M. peltata* leaf extract was carried out on male rabbits based on a modified method [13]. This study was approved by the Research Ethics Committee to protect the rights and welfare of the test animals (1827/UN29.20.1.2/PG/2020). The process of acclimatization in rabbits was carried out through adaptation to environmental conditions to relieve stress on test animals.

After 21 days of testing, among the test groups, the ethanol extract of *M. peltata* leaves at a concentration of 30% showed the highest hair growth yield of 1.33 ± 0.2 mm, while the group treated with 10% extract showed the least hair growth, that is, 1.06 ± 0.3 mm. Figure 1 shows that hair growth in the normal and negative controls was not too fast compared to that in the positive control and the groups treated with *M. peltata* leaf extract.

### 3.2. Hair Growth Activity of VLC Fractions of Purified *M. peltata* Leaf Extract

**Figure 2:**
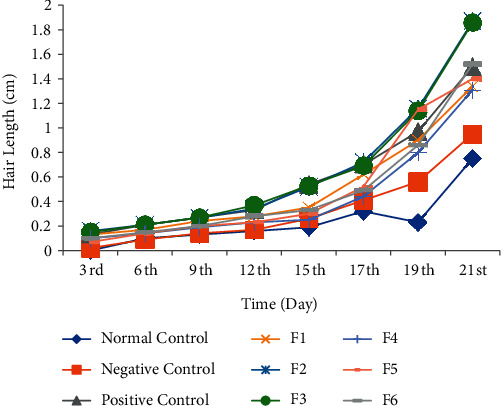
Test of liquid vacuum chromatography fractions of *M. peltata* leaf ethyl acetate extract.

In the *in vivo* hair growth activity test, the five *M. peltata* leaf extract fractions obtained by VLC (F1–F5) at a concentration of 30%, a positive control of 2% minoxidil, a negative control of Na-CMC, and a normal control were tested for 21 days of observation in test animals. The results showed that the average hair growth level in the positive control (minoxidil) was 1.50 ± 0.32 mg/dL, in the normal control group, it was 0.75 ± 0.28 mg/dL, in the negative control, it was 0.95 ± 0.04 mg/dL, and in fraction F1 to fraction F5 groups, it was 1.34 ± 0.63, 1.87 ± 0.32, 1.86 ± 0.48, 1.31 ± 0.73 mg/dL, and 1.40 ± 0.61 mg/dL, respectively. The graph of the results of measuring the average hair growth in rabbits treated with the fractions of purified *M. peltata* leaf extract (F1–F5) is seen in Figure 2.

The results of the analysis for 21 days showed that the best hair growth was observed in the rabbits treated with fractions F2 and F3 compared to positive controls, with the average hair length on day 21 being 1.87 ± 0.32 and 1.86 ± 0.48 mm, respectively.

### 3.3. Compounds Identified Using LC-MS/MS

LC-MS/MS analysis was used to determine the secondary metabolite profile of the *M. peltata* leaf extract fractions obtained by VLC (F1–F5).

The content of compounds is indicated by chromatogram peaks with different molecular weight differences. The MS spectra were interpreted using a spectrum database for organic compounds in the SDBS application. The content of compounds examined by LC/MS-MS can be seen in Table 2.

**Figure 3:**
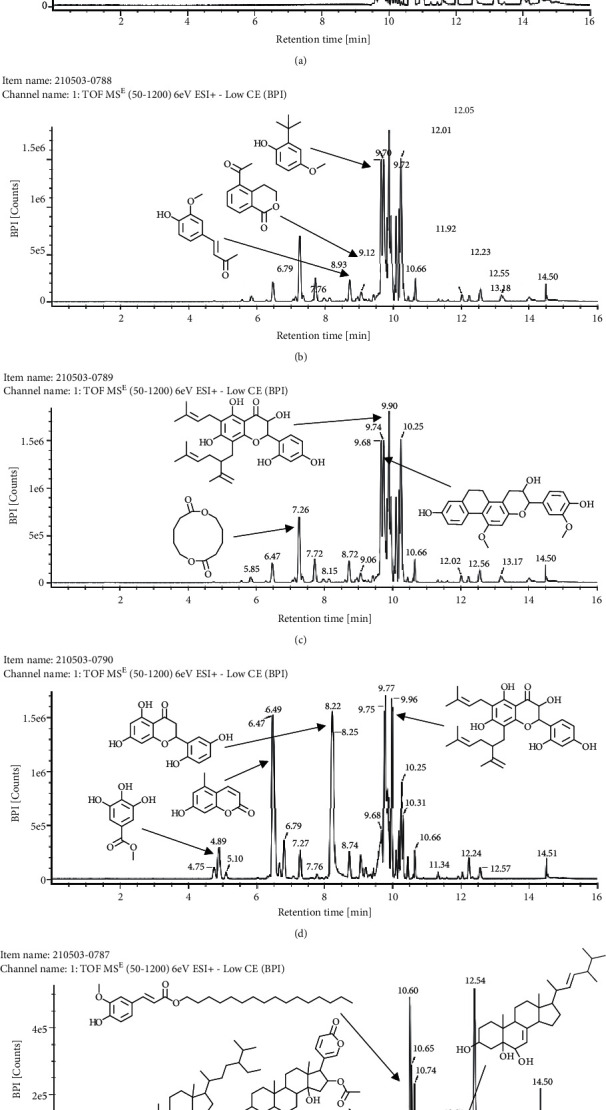
Mass spectrums of the compounds from *M. peltata* leaves fractions (F1–F5).

Based on the results of the analysis in Table 2, several compounds found were the same in each fraction and in accordance with the research of [10, 11] flavonoids, terpenoids, and phenylpropanoids. The analysis of the data on the spectrum of the compounds contained is seen in Figure 3.

The LC-MS/MS successfully screened and identified several compounds that were selected based on the similarity percentage of their retardation time (Rt) and molecular mass with the database from UNIFI 1.8 software. LC-MS/MS analysis of the VLC fraction of *M. peltata* leaf extract revealed different types of compound groups based on the molecular structure of the compounds analyzed. In fractions 1 and 3, there were ester group compounds, namely, (E)-hexadecyl-ferulate and digiprolactone derived from carboxylic acid derivative compounds that undergo oxidation reactions in the hydroxyl group, where the carboxylic acid group is in the form of phenylpropanoid, which is a derivative compound of cinnamic acid. Cinnamic acid is derived from L-phenylalanine through a deamination reaction. Hydrogenation of cinnamic acid produces p-coumaric acid, which is an intermediate compound for the formation of phenylpropanoid derivatives, namely, p-hydroxy sinapyl alcohol, coniferyl alcohol, and sinapyl alcohol. Furthermore, in fraction 1, there were also steroid group compounds such as bufotalinin, cerevisterol, and stigmastan-3,6-dione. Steroid compounds are derived from modified triterpene forms, so that the constituent units are isoprene, namely, isopentenyl pyrophosphate (IPP) and dimethylallyl pyrophosphate (DMAPP). IPP and DMAPP are biosynthesized by the body from acetyl coenzyme A, a C-2 product of the release of CO_2_ by pyruvate in the metabolic pathway, via the mevalonic acid or deoxyxylulose phosphate pathway. The units of IPP and DMAPP react to elongate the chain to form C-15, called farnesyl. Two farnesyl pyrophosphate (FPP) molecules join tails to form squalene. Squalene is oxidized to form an epoxide, allowing cyclization to form lanosterol.

In fraction 4, there were coumarin group compounds, namely, 7-hydroxy-5-methoxycoumarin, which is biosynthetically derived from coumarin formation; it has similarities with chlorogenic acid and rosmarinic acid and can be formed from aromatic amino acids such as L-tyrosine. Modification of L-tyrosine will produce 4-hydroxyphenylpyruvic acid, and then, through several reaction steps, such as reduction and dehydration, it produces rosmarinic acid, while chlorogenic acid can be formed through a substitution reaction between caffeoyl-CoA and quinic acid. Aromatic polyketide compounds from the LC-MS/MS analysis were found in fraction 2 (3-tert-butyl-4-methoxyphenol, erythrocentaurin, and trans-ferulaldehyde) and fraction 4 (methyl gallate). Polyketide compounds are formed from poly-*β*-ketoesters, which are formed from four acetate units (one acetate as a starter and three malonic units as an extension) and can bend in two ways of ionization to produce aromatic polyketides. In the process of fatty acid biosynthesis, malonyl-CoA and acetyl-CoA are first converted into aryl carrier protein (ACP) and thioester. Claisen condensation of the two unions forms *β*-ketoacyl–ACP. Flavonoid compounds were found in fraction 3 (kushenol M and shanciol B), fraction 4 (5,7,2′,5′-tetrahydroxy-flavone and kushenol M), and fraction 5 (5,7,2′,5′-tetrahydroxy-flavones, epigallocatechin(4*β*,8)-gallocatechin, kaempferol-3-O-*β*-D-glucopyranoside, kaempferol-7-O-*α*-L-rhamnoside, and tiliroside); their formation involves dehydrogenation, elimination, alkene reduction, and hydration reactions [26].

### 3.4. Preparation of Protein Receptor

**Figure 4:**
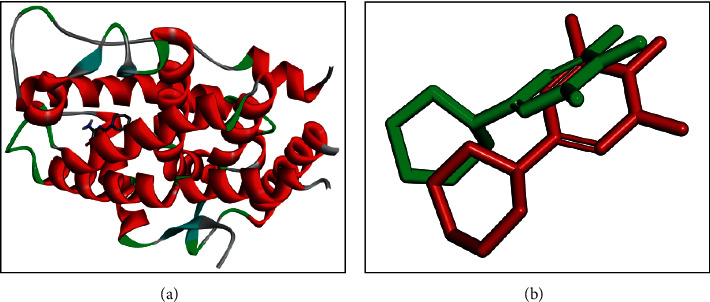
Structure of (a) androgen receptor (4K7A) and (b) overlay of docked pose of minoxidil with that of the cocrystallized ligand of 4K7A.

The androgen receptor (4K7A) interacts with natural ligands via chemical bonds. The positions of the natural ligand (minoxidil) that are overlaid as a result of redocking on the androgen receptor are depicted in Figure 4. Natural ligands that interacted with the androgen receptor (4K7A) were separated using Discovery Studio Visualizer software.

Androgen receptors are activated by binding interactions with androgen. Under physiological conditions, these hormones are present in all sexes, but their concentration is highest in men, while in women they are a precursor of female sex hormones, which are converted into oestrogen [19]. This study uses the androgen receptor model PDB code 4K7A. The ligand-binding domain of the androgen receptor is encoded by residues 690–919 (unitprot.org).

In the docking process, a ligand is needed. The ligand selection process used in the target protein tethering process is based on the initial selection results based on Lipinski's Rule of Five. The preparation of natural ligands on the androgen receptor (4K7A) resulted in 2.28 and 2.90 Ǻ hydrogen bonds for the interaction of minoxidil with SER^865^ and GLU^793^ of the androgen receptor with a binding energy of −4.8 kcal/mol. A smaller value of ΔG indicates a more stable bond.

### 3.5. Validation of Molecular Docking Method

Analysis of the bonds formed between minoxidil and the androgen receptor (4K7A) was performed using Discovery Studio Visualizer software. The results of the analysis of the bonds formed are shown in Table 3.

**Figure 5:**
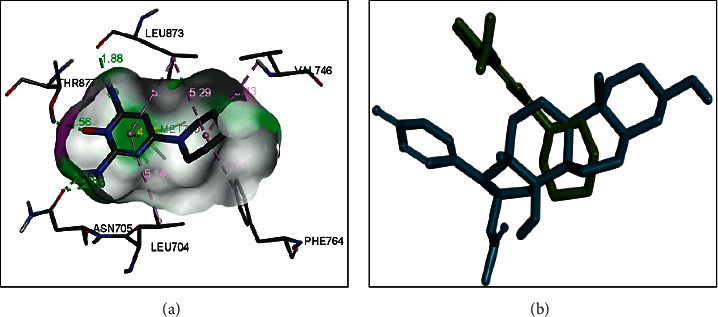
(a) Visualization of interactions between minoxidil and androgen receptor (4K7A) and (b) overlay of the docked poses of the test compounds on that of minoxidil.

The interaction between minoxidil and the receptor occurs by hydrogen bonds derived from the amino acids SER^865^ and GLU^793^. The closest residues in the minoxidil–androgen receptor complex are LEU^862^, LYS^861^, and TYR^857^ (Figure 5).

Molecular validation of the docking was carried out by redocking minoxidil to the target protein. The minoxidil in position is repositioned at the initial coordinates to the androgen receptor (4K7A) at a resolution of 2.44 Ǻ. The root mean square deviation (RMSD) value of minoxidil redocking was 2.31, and the bond energy obtained was −4.8 kcal/mol. According to [13], an RMSD value of 3 Å and similar binding energy to the redocking results indicate that the interaction between the ligand and the receptor is in a low energy state; thus, the molecule will be more stable. The hydrogen bonds formed between SER^865^ and GLU^793^ and the -NH^2^ and –NO functional groups of minoxidil showed values of 2.28 and 2.90 Ǻ, respectively. The interaction that occurs with SER^865^ and GLU^793^ is thought to play an important role in the androgen receptor ligand-binding domain.

### 3.6. Docking Simulation of Minoxidil, Finasteride, and Test Ligands (Phytocompounds in Purified *M. peltata* Leaf Extract)

The docking simulation was carried out by setting the coordinates in accordance with the position of the interaction between minoxidil and the androgen receptor (4K7A) using the AutoDock Tools 1.5.6 program. This analysis was conducted to determine the value of bond energy and the formation of hydrogen bonds with minoxidil, finasteride, and the 17 test ligands with known structure from the LC-MS. Compounds derived from LC-MS with unknown structure were not included in this study. The results of the docking simulation are shown in Table 4.

**Figure 6:**
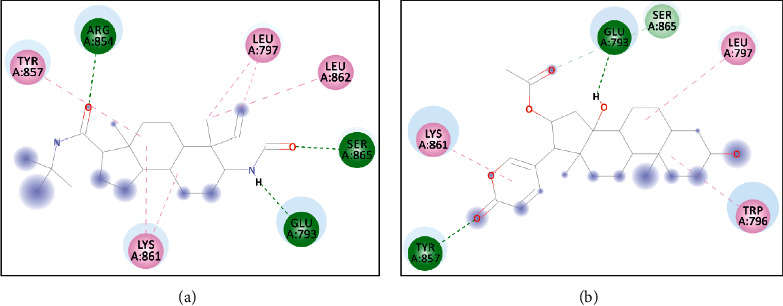
Visualization of molecular docking between the androgen receptor and (a) the reference ligand finasteride and (b) bufotalinin.

A description of the interaction of the docking results was obtained for the standard compound finasteride and derived from the VLC fractions of *M. peltata* leaf extract on the androgen receptor (4K7A), and bufotalinin has the best binding energy value to the androgen receptor (4K7A) compared to the other compounds. The docking result visualization is shown in Figure 6.

Docking is a bonding interaction between ligands and proteins that is used to predict the position and orientation of the ligand when it is bound to the protein receptor. The docking process will produce a bond energy (ΔG), which is a parameter of the conformational stability between the ligand and the androgen receptor [27]. Based on the results of docking minoxidil and to the androgen receptor, the binding energy value (ΔG) for minoxidil was −4.8 kcal/mol (Table 2) and that for (bufotalinin) was −5.99 kcal/mol.

Hydrogen bonds formed between minoxidil and androgen receptors occur in interactions with the amino acids SER^865^ and GLU^793^, and other closest amino acid residues are LEU^862^, LYS^861^, and TYR^857^. Based on the docking results, it had a lower binding energy value than minoxidil. Its low binding energy is influenced by the presence of the same amino acid in minoxidil and also in the standard compound finasteride. The amino acid residues were LYS^861^ and LEU^797^ (Figure 5). The binding energy of and finasteride is higher than that of minoxidil; one of the factors causing the binding energy of minoxidil to be lower is the influence of hydrogen bonds formed with amino acids SER^865^ and GLU^793^ with interaction values of 2.28 and 2.90 Ǻ, respectively. The interaction occurs in the side chain with the -NH2 and -NO functional groups, whereas in finasteride the hydrogen bonds formed on the amino acids ARG^854^, GLU^793^, and SER^865^ have interaction values of 2.86, 1.88, and 2.13 Ǻ on the side chains of the -NH and -CO functional groups. Furthermore, it forms hydrogen bonds with amino acids TYR^857^ and GLU^793^ with values of 2.03 and 2.36 Ǻ, respectively, with interactions at -CO and -OH. Hydrophobic interactions play a role in determining the stability of ligands with androgen receptors. Hydrophobic interactions are interactions that avoid the liquid environment and tend to cluster inside the globular structure of the protein [28]. The formation of hydrophobic bonds can minimize the interaction of nonpolar residues with water. In minoxidil, hydrophobic interactions with ligands occurred at residues LEU^862^, LYS^861^, and TYR^857^, while involved residues were LYS^861^, TRP^796^, LEU^797^, and SER^865^. The residues involved in hydrophobic interactions are residues of nonpolar amino acids that tend to form groups in the interior of the protein.

The interactions that occur in each ligand and amino acid residue involve several residues that are important in the interaction of ligands and macromolecules. In Table 3, residues SER^865^, LYS^861^, GLU^793^, and TRP^796^ always appear in every ligand–receptor interaction in the form of hydrogen bonds or hydrophobic interactions, so that these residues are predicted to play an important role in the binding site area of the androgen receptor. GLU is found in hydrogen bonds from androgen receptor interactions, but based on simulation results on reference ligands, it is shown that GLU is predicted to play an important role in the binding site area of receptor proteins. Protein binding sites are areas of protein binding to molecules and ions (ligands) that affect the conformation and function of the protein. The binding site area involves residues of amino acids that have an important role in binding to the ligand. The interactions that occur between the ligands and the amino acid residues are formed as hydrogen bonds, hydrophobic interactions, and electrostatic interactions.

### 3.7. MD Simulation

**Figure 7:**
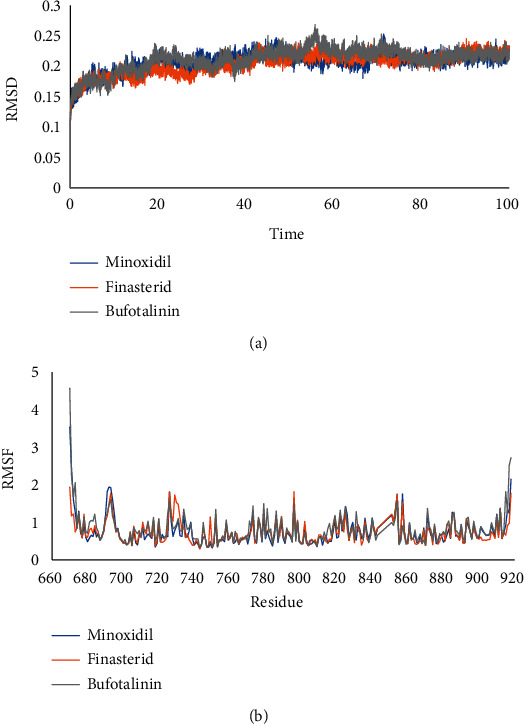
RMSD (a) and RMSF (b) value of complexes of minoxidil–androgen (blue), finasteride–androgen (orange), and bufotalinin–androgen (grey).

MD simulation was carried out on the compound bufotalinin, which had the lowest binding energy value, the natural ligand minoxidil, and the reference compound finasteride. RMSD and root mean square fluctuation (RMSF) analyses of ligand–receptor complexes were carried out during a 100 ns MD simulation using GROMACS 2016. The stability of the system during the simulation was successfully measured through RMSD and RMSF (Figure 7).

RMSD analysis was used to assess the stability of the complex over time, while RMSF analysis assessed the stability per amino acid. Bufotalinin, with the best docking score of the metabolites, was simulated with MD and its complex stability compared to that of two reference ligands, namely, minoxidil and finasteride, which are androgen receptor blockers. Bufotalinin in complex with the androgen receptor showed the same high fluctuations as minoxidil and finasteride. Meanwhile, the average RMSD fluctuations for each system, namely, minoxidil, finasteride, and bufotalinin, were 0.206, 0.203, and 0.211, respectively. The average RMSD showed that bufotalinin had the lowest fluctuation compared to the reference ligand, which indicates that the ligand has reached a stable conformation that binds to the protein [29]. The amino acid fluctuations of the two receptor complex systems calculated by RMSF showed the same pattern in all regions. Residues 693, 726, 729, and 796 on the androgen receptor showed higher fluctuations than the other residues. These residues are the amino acids responsible for the loop region in the protein structure.

**Figure 8:**
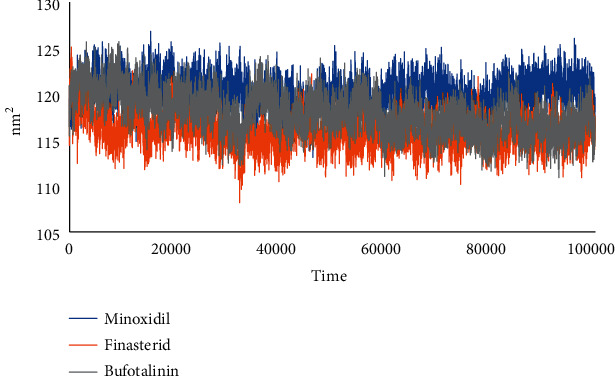
SASA plot of complexes of minoxidil–androgen (blue), finasteride–androgen (orange), and bufotalinin−androgen (grey).

The solvent-accessible surface area (SASA) was identified to predict the conformational changes of proteins during simulations that were accessible to water molecules. This analysis, correlating with RMSD values, showed that bufotalinin had better stability at the androgen receptor than minoxidil, as shown in Figure 8.

SASA was analyzed for 100 ns of simulated MD trajectory, as shown in Figure 8. The SASA of the androgen receptor–ligand complex on the graph for bufotalinin showed similar fluctuations, which were higher than those for minoxidil and finasteride; the average values for minoxidil, finasteride, and bufotalinin were 120.13, 115.93, and 117.96 nm^2^, respectively. A low SASA value indicates an increasingly stable complex system [29]. This analysis correlated with that of the RMSD value, which indicated that bufotalinin had better stability at the androgen receptor than minoxidil and finasteride.

**Figure 9:**
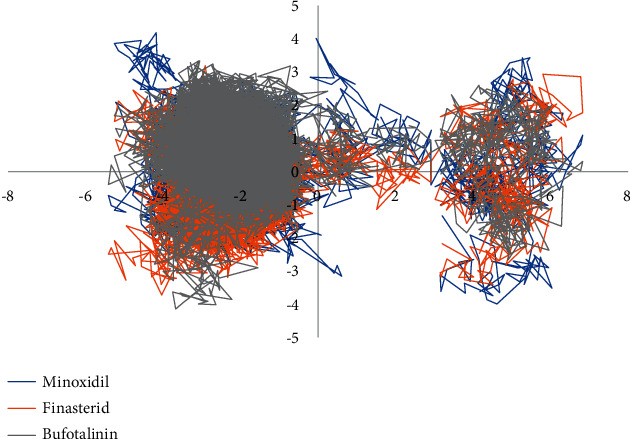
PCA plot of complexes of minoxidil–androgen (blue), finasteride–androgen (orange), and bufotalinin–androgen (grey) represented by 2D projection of trajectory motion during MD simulations.

Principal component analysis (PCA) was used to analyze significant fluctuations in the protein–ligand complex. The direction and amplitude of the eigenvectors, which are responsible for the motion and complex dynamics, are analyzed in a 2D projection of trajectory plotting. The plot shows that bufotalinin was stable for the 100 ns simulation of binding to the androgen receptor (Figure 9).

The selection of the two main components, namely, PC1 and PC2 in Figure 9, shows the projection of two eigenvectors for finasteride, minoxidil, and bufotalinin in complex with the androgen. Less space occupied by a cluster indicates a more stable complex, while one occupying more space indicates a less stable protein–ligand complex [29]. From the 2D eigenvector plots, it can be seen that bufotalinin occupies less phase space than the minoxidil and finasteride patterns. The plot shows that bufotalinin was stable for 100 ns of simulation of androgen receptor binding.

### 3.8. ADME-Tox Prediction

Pharmacokinetic analysis and ADME-Tox prediction were carried out to determine compounds that could act as drugs. ADME-Tox analysis was used to predict compounds that have antialopecia activity; the predicted values are shown in Tables 5 and 6.

Model name and units: 1: water solubility (numeric log mol/L), 2: Caco-2 permeability (numeric log Papp in 10^−6^ cm/s), 3: intestinal absorption (human) (numeric [% absorbed]), 4: skin permeability (numeric [log Kp]), 5: VDSS (human) (numeric [log L/kg]), 6: fraction unbound (human) (numeric [Fu]), 7: BBB permeability (numeric [log BB]), 8: CNS permeability (numeric [log PS]).

Model name and units: 9: CYP3A4 substrate (yes/no), 10: CYP2C9 inhibitor (yes/no), 11: total clearance (log mL/min/kg), 12: AMES toxicity (yes/no), 13: maximum tolerated dose (human) (log mg/kg/day), 14: hERG I inhibitor (yes/no), 15: hERG II inhibitor (yes/no), 16: oral rat acute toxicity (LD) (mol/kg), 17: hepatotoxicity (yes/no), 18: skin sensitization (yes/no), 19: minnow toxicity (log mM).

The percentage of plasma protein binding (%PB) is an important pharmacokinetic factor determining dosing regimen (frequency) but not daily dosing [30]. Distribution occurs due to a shift in plasma protein binding. Drug interactions involving the distribution process will be clinically relevant if the drug has a %PB > 85%, a small volume of distribution (VD), and a narrow safe limit. Minoxidil has a PB value of 99% and a Vd of 0.142. It has a PB of 91% and a VD of 0.037. Based on these results, they have good plasma protein binding attributes.

The prediction of the distribution value with the pkCSM tool can predict VDSS, the reaction volume at the total required dose in blood plasma. The higher the value of VDSS, the more the drug reserves distributed to the tissues from the plasma. [31] The value of VDSS can be accepted with a low volume of distribution if the log value of VDSS < −0.15 and > 0.45. The results of the examination with pkCSM showed that the log VDSS value of minoxidil was 0.142, while it had a log VDSS value of 0.319. Based on the definition of an acceptable value of VDSS as defined by [32], it is better than minoxidil.

The Caco-2 cell model and the Madin–Darby renal cell model (MDCK) are *in vitro* models that can be used to predict oral drug absorption. Caco-2 single-cell monolayer permeability is an *in vitro* model of the intestinal mucosa used to predict the absorption of orally administered drugs. Polar bonds do not give a good Caco-2 permeability value, but a more polar surface area will provide stronger hydrogen bond interactions between Caco-2 cells and the drug [33]. Compounds are said to have high Caco-2 permeability if Papp >8 × 10^6^ cm/s [32]. Based on the results of the pkCSM analysis, a value > 0.90 is considered a high log Papp value and indicates a permeable compound. In this study, minoxidil and had Papp values of 0.653 and 0.693, respectively. This indicates that it has a greater Caco-2 permeability than minoxidil.

## 4. Conclusions

Based on the results of *in vivo* tests on rabbit hair growth activity, ethanol extract of *M. peltata* leaves at a concentration of 30% gave excellent hair growth effectiveness and rabbit hair growth activity from the vacuum liquid chromatography fraction of purified extract of *M. peltata* leaves using a concentration of 30% gave growth activity, the best in the F2 and F3 fractions. Furthermore, it is identified using Liquid Chromatography-Mass Spectrometry (LC-MS) on the vacuum liquid chromatography fraction of *M. peltata* leaves obtained as much as (A-E) fraction containing steroid compounds, flavonoids, coumarins, and aromatic polyketides. *In in silico* analysis using the androgen receptor (4K7A) for minoxidil, the comparison compound finasteride, and the compound assay of *M. peltata* leaf vacuum liquid chromatography fraction, bufotalinin has the best binding energy, which is higher than minoxidil as a natural ligand. Molecular dynamics simulation showed that bufotalinin has RMSD, RMSF, SASA, and PCA values, which are very much compared to the natural ligand minoxidil. ADME-Tox analysis on minoxidil showed a good value profile. Therefore, it has the potential to be an antialopecia drug. Further research is needed to isolate the biological compounds contained in *M. peltata* leaves and perform *in vitro* and *in vivo* tests.

## Figures and Tables

**Table 1:** Two-dimensional structure of minoxidil, finasteride, and assay ligands from purified *M. peltata* leaf extract.

No.	IUPAC name	Structure
1	Compound 1 (E)-Hexadecyl-ferulate	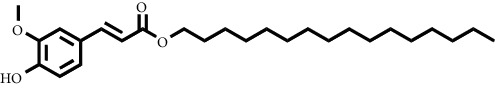

2	Compound 2 Bufotalinin	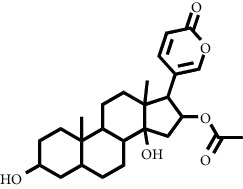

3	Compound 3 Cerevisterol	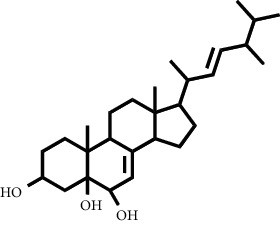

4	Compound 4 Stigmastan-3,6-dione	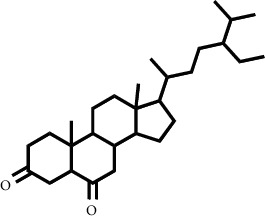

5	Compound 5 3-Tert-butyl-4-methoxyphenol	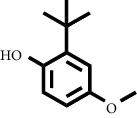

6	Compound 6 Erythrocentaurin	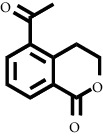

7	Compound 7 Trans-ferulaldehyde	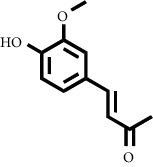

8	Compound 8 Digiprolactone	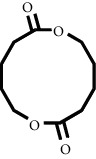

9	Compound 9 Kushenol M	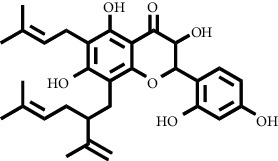

10	Compound 10 Shanciol B	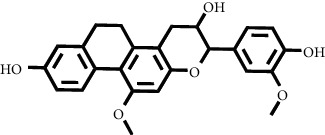

11	Compound 11 5,7,2,5′-Tetrahydroxy-flavone	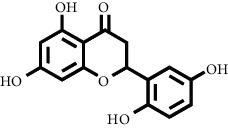

12	Compound 12 7-Hydroxy-5-methoxycoumarin	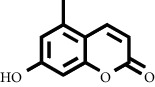

13	Compound 13 Methyl gallate	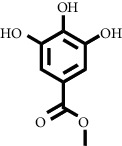

14	Compound 14 Epigallocatechin(4*β*,8)-gallocatechin	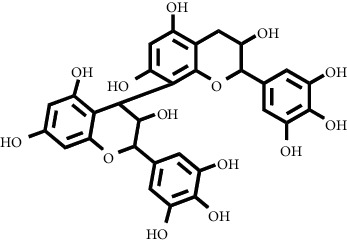

15	Compound 15 Kaempferol-3-O-*β*-D-glucopyranoside	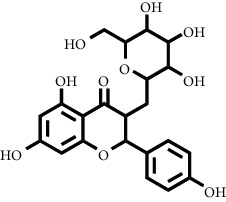

16	Compound 16 Kaempferol-7-O-*α*-L-rhamnoside	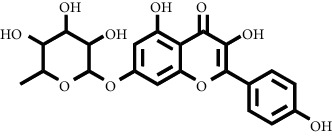

17	Compound 17 Tiliroside	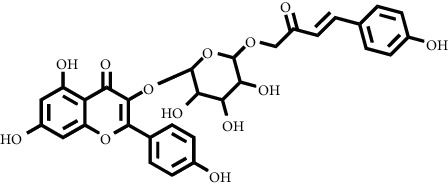

18	Reference ligand Finasteride	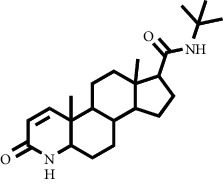

19	Natural ligand Minoxidil	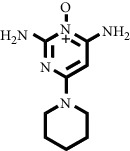

**Table 2:** Identification of compounds in *M. peltata* leaf extract fractions using liquid chromatography–mass spectrometry (LC-MS/MS).

Fraction	Compound	Observed Rt (min)	Observed *m/z*
F1	(E)-Hexadecyl-ferulate	10.74	441.2979
	Bufotalinin	9.85	415.2121
	Cerevisterol	12.01	453.3342
	Stigmastan-3,6-dione	10.96	429.3725
	Candidate mass C_35_H_66_O_6_	12.02	605.4751

F2	3-Tert-butyl-4-methoxyphenol	9.70	181.1223
	Erythrocentaurin	9.12	177.0545
	Trans-ferulaldehyde	8.93	179.0702
	Candidate mass C_54_H_78_O_10_	12.04	887.5681
	Candidate mass C_54_H_78_O_11_	11.92	903.5632

F3	Digiprolactone	7.26	197.1173
	Kushenol M	9.92	509.2534
	Shanciol B	9.67	423.1796
	Candidate mass C_26_H_48_O_14_	10.25	607.2926
	Candidate mass C_26_H_46_O_15_	10.11	621.2721

F4	5,7,2′,5′-Tetrahydroxy-flavone	8.22	287.0554
	7-Hydroxy-5-methoxycoumarin	6.49	193.0497
	Kushenol M	9.77	509.2535
	Methyl gallate	4.89	185.0444
	Candidate mass C_30_H_31_O_7_	9.96	505.2228

F5	5,7,2′,5′-Tetrahydroxy-flavone	8.23	287.0552
	Epigallocatechin (4*β*,8)-gallocatechin	7.48	611.1406
	Kaempferol-3-O-*β*-D-glucopyranoside	6.23	449.1082
	Kaempferol-7-O-*α*-L-rhamnoside	6.04	433.1132
	Tiliroside	8.07	595.1451

**Table 3:** Validation results for the molecular docking method.

Protein	Compound	Binding energy (kcal/mol)	RMSD	Hydrogen bond distance (Ǻ)	Amino acids that bind	Binding functional group	Nearest residues
4K7A	Minoxidil	−4.8	2.31	2.28 and 2.90	SER^865^ and GLU^793^	-NH_2_ and -NO	LEU^862^, LYS^861^, TYR^857^

RMSD: root mean square deviation.

**Table 4:** Docking simulation results.

Compound	Binding energy (kcal/mol)	Hydrogen bond distance (Ǻ)	Hydrogen bonds	Nearest amino acid residue (s)
Minoxidil	−4.8	2.28 and 2.90	SER^865^ and GLU^793^	LEU^862^, LYS^861^, TYR^857^
Finasteride	−6.03	2.86, 1.88 and 2.13	ARG^854^, GLU^793^ and SER^865^	LYS^861^, LEU^797^
(E)-Hexadecyl-ferulate	−1.89	—	—	GLU^793^, TRP^796^, HIS^789^
Bufotalinin	−5.99	2.03 and 2.36	TYR^857^ and GLU^793^	LYS^861^, TRP^796^, LEU^797^, SER^865^
Cerevisterol	−5.38	—	—	LEU^862^, LYS^861^, TYR^857^, LEU^797^, PRO^868^
Stigmastan-3,6-dione	−5.65	—	—	LYS^861^, LEU^862^, LEU^797^
3-Tert-butyl-4-methoxyphenol	−4.81	3.02, 2.24 and 2.01	ARG^786^, GLU^793^ and SER^865^	LEU^790^, HIS^789^
Erythrocentaurin	−4.81	2.17	SER^865^	LEU^862^, LYS^861^, LEU^797^
Trans-ferulaldehyde	−4.11	3.09 and 1.97	GLU^793^ and GLN^858^	LYS^861^, LEU^862^
Digiprolactone	−4.98	2.13	ARG^786^	GLU^793^
Kushenol M	−4.02	2.19 and 2.20	HIS^789^ and GLU^793^	TYR^857^, ARG^854^, LEU^797^, LYS^861^
Shanciol B	−4.79	2.67 and 2.17	LYS^861^ and GLN^858^	GLU^793^, TRP^796^, LEU^797^
5,7,2′,5′-Tetrahydroxy-flavone	−4.75	1.92, 2.37 and 1.77	TRP^796^, GLU^793^ and GLN^858^	LEU^797^
7-Hydroxy-5-methoxycoumarin	−4.31	2.12	GLN^858^	LYS^861^, LEU^862^
Methyl gallate	−3.58	−1.86 and 2.19	GLU^793^ and GLN^858^	LEU^797^, LYS^861^
Epigallocatechin (4*β*,8)-gallocatechin	−3.93	2.01, 2.24 and 2.18	LYS^861^, GLU^793^ and TRP^796^	LEU^797^, HIS^789^
Kaempferol-3-O-*β*-D-glucopyranoside	−4.14	2.37, 2.30, 2.11, 1.76 and 2.04	HIS^789^, GLN^85^, TYR^857^, SER^865^ and GLU^793^	LYS^861^
Kaempferol-7-O-*α*-L-rhamnoside	−4.52	2.13, 2.86 and 2.50	ARG^854^, TYR^857^ and GLU^793^	LYS^861^
Tiliroside	−3.68	2.07 and 2.36	TRP^796^ and GLN^858^	TYR^857^, LEU^797^

**Table 5:** Absorption and distribution prediction values.

Compound	Absorption				Distribution			
	1	2	3	4	5	6	7	8
Minoxidil	−2.871	0.653	94.641	−2.798	0.142	0.773	−0.951	−3.471
Finasteride	−5.148	1.269	93.742	−3.463	−0.185	0.01	−0.18	−1.821
Bufotalinin	−4.862	0.693	97.723	−3.588	0.037	0.034	−0.485	−2.107
3-Tert-butyl-4-methoxyphenol	−2.287	1.698	92.604	−1.802	0.319	0.307	0.353	−1.559
Erythrocentaurin	−1.804	1.263	88.581	−2.539	−0.148	0.372	−0.161	−2.221
Digiprolactone	−1.093	1.251	98.115	−3.515	−0.311	0.653	−0.281	−2.908

**Table 6:** Metabolism, excretion, and toxicity prediction results.

Compound	Metabolism		Excretion	Toxicity							
	9	10	11	12	13	14	15	16	17	18	19
Minoxidil	No	No	0.275	No	−0.359	No	No	2.286	Yes	No	3.516
Finasteride	Yes	Yes	0.38	No	−1.355	No	No	2.424	Yes	No	0.638
Bufotalinin	Yes	No	0.42	No	−0.837	No	No	2.865	Yes	No	0.672
3-Tert-butyl-4-methoxyphenol	No	No	0.234	No	0.595	No	No	2.075	Yes	Yes	0.816
Erythrocentaurin	No	No	0.685	No	1.06	No	No	1.791	Yes	No	1.831
Digiprolactone	No	No	0.605	No	0.816	No	No	2.03	No	Yes	2.621

## Data Availability

Data is available upon request.

## References

[1] Obasi C. J., Obasi I. S., Okafor U. C., Uzoka I. S. (2018). Comparison of anti-dandruff activity of synthetic shampoos and crude plant extracts on dandruff causing isolates. *Journal Biotechnol. Biochem.*.

[2] Semwal D., Kotiyal R., Chauhan A. (2015). Alopecia and the herbal drugs: an overview of the current status. *Advances in Biomedicine and Pharmacy*.

[3] Guo E. L., Katta R. (2017). Diet and hair loss: effects of nutrient deficiency and supplement use. *Dermatology Practical and Conceptual*.

[4] Ruslin, Sahidin I. (2008). Identification and determination of traditional medicinal plants in Southeast Sulawesi community at Arboretum Prof. Mahmud Hamundu Haluoleo University. *Majalah Farmasi Indonesia*.

[5] Honesty R. (2012). *Antibacterial Activity Test of Lambuang Aka Leaf Fraction (Merremia Peltata (L.) Merr.)*.

[6] Perez K. J. B., Jose M. A. I., Aranico E., Madamba M. R. S. B. (2015). Phytochemical and antibacterial properties of the ethanolic leaf extract of *Merremia peltata* L. Merr. and Rubus spp. *Advances in Environmental Biology*.

[7] Luo J., Chen M., Liu Y. (2018). Nature-derived lignan compound VB-1 exerts hair growth-promoting effects by augmenting Wnt/*β*-Catenin signaling in human dermal papilla cells. *PeerJ*.

[8] Mustarichie R., Wicaksono I. A., Gozali D. (2017). Anti-alopecia activity of DADAP (*Erythrina variegata* L.) leaves ethanol extract. *Journal of Pharmaceutical Sciences*.

[9] Kaushik R., Gupta D., Yadav R. (2011). Alopecia: herbal remedies. *International Journal of Pharmaceutical Sciences and Research*.

[10] Semwal B. C., Agrawal K. K., Singh K., Tandon S., Sharma S. (2011). Alopecia: switch to herbal medicine. *Journal of Pharmaceutical Research and Opinion*.

[11] Badolati N., Sommella E., Riccio G. (2018). Annurca apple polyphenols ignite keratin production in hair follicles by inhibiting the pentose phosphate pathway and amino acid oxidation. *Nutrients*.

[12] Hasanah A., Abdurrahman S., Ruslin R., Mustarichie R. (2021). Molecular docking studies and ADME-Tox prediction of phytocompounds from *Merremia peltata* as a potential anti-alopecia treatment. *Journal of Advanced Pharmaceutical Technology & Research*.

[13] Wright D. B. (2006). Comparing groups in a before-after design: when *t*-test and ANCOVA produce different results. *British Journal of Educational Psychology*.

[14] Juliawaty L. D., Ra’idah P. N., Abdurrahman S. (2020). 5, 6-Dihydro-*α*-pyrones from the leaves of *Cryptocarya pulchinervia* (Lauraceae). *Journal of Natural Medicines*.

[15] Tanaka S., Saito M., Tabata M. (1980). Bioassay of crude drugs for hair growth promoting activity in mice by a new simple method. *Planta Medica*.

[16] Federer W. (1963). *Experimental Design, Theory, and Application*.

[17] Hanafi C. I., Henny R., Lilis S., Achmad N. R., Supriyono (2018). Phytochemical screening, LC-S studies and antidiabetic potential of methanol extract of seed shells of *Archidendron bubulinum* (Jack) I.C. Nielson (*Julang jaling*) from Lampung, Indonesia. *Pharmacognosy Journal*.

[18] Jain A. N., Nicholls A. (2008). Recommendations for evaluation of computational methods. *Journal of Computer-Aided Molecular Design*.

[19] Guay A. T. (2001). Advances in the management of androgen deficiency in women. *Medical Aspects of Human Sexuality*.

[20] Culig Z., Klocker H., Bartsch G., Hobisch A. (2002). Androgen receptors in prostate cancer. *Endocrine-Related Cancer*.

[21] Abraham M. J., Murtola T., Schulz R. (2015). GROMACS: high performance molecular simulations through multi-level parallelism from laptops to supercomputers. *SoftwareX*.

[22] Essmann U. L., Perera L., Berkowitz M. L., Darden T., Lee H., Pedersen L. G. (1995). A smooth particle mesh Ewald method. *The Journal of Chemical Physics*.

[23] Alan W. S. d. S., Wim F. V. (2012). ACPYPE-AnteChamber PYthon parser interfacE. *BMC Research Notes*.

[24] Mark P., Nilsson L. (2001). Structure and dynamics of the TIP3P, SPC, and SPC/E water models at 298 K. *The Journal of Physical Chemistry A*.

[25] Lipinski C. A., Lombardo F., Dominy B. W., Feeney P. J. (2001). Experimental and computational approaches to estimate solubility and permeability in drug discovery and development settings 1PII of original article: S0169-409X(96)00423-1. The article was originally published in Advanced Drug Delivery Reviews 23 (1997) 3–25. 1. *Advanced Drug Delivery Reviews*.

[26] Dewick P. M. (2002). *Medicinal Natural Products: A Biosynthetic Approach*.

[27] Girija C. R., Karunakar P., Poojari C. S., Begum N. S., Syed A. A. (2010). Molecular docking studies of curcumin derivatives with multiple protein targets for procarcinogen activating enzyme inhibition. *Journal of Proteomics & Bioinformatics*.

[28] Lins L., Brasseur R. (1995). The hydrophobic effect in protein folding. *The FASEB Journal*.

[29] Pitaloka D. A. E., Ramadhan D. S. F., Arfan, Chaidir L., Fakih T. M. (2021). Docking-based virtual screening and molecular dynamics simulations of quercetin analogs as enoyl-acyl carrier protein reductase (InhA) inhibitors of *Mycobacterium tuberculosis*. *Scientia Pharmaceutica*.

[30] Mannhold R. (2008). *Molecular Drug Properties. Measurement and Prediction*.

[31] Bowers L. D. (1989). High-performance liquid chromatography/mass spectrometry: state of the art for the drug analysis laboratory. *Clinical Chemistry*.

[32] Pires D. E. V., Blundell T. L., Ascher D. B. (2015). pkCSM: predicting small-molecule pharmacokinetic and toxicity properties using graph-based signatures. *Journal of Medicinal Chemistry*.

[33] Hou T. J., Zhang W., Xia K., Qiao X. B., Xu X. J. (2004). ADME evaluation in drug discovery. 5. Correlation of Caco-2 permeation with simple molecular properties. *Journal of Chemical Information and Computer Sciences*.

